# EXPLORING THE COMPLEXITY OF LUCID EPISODES IN DEMENTIA: PERSPECTIVES FROM MULTIDISCIPLINARY CLINICAL EXPERTS

**DOI:** 10.1093/geroni/igad104.3333

**Published:** 2023-12-21

**Authors:** Lauren Bangerter, Joan Griffin, Kyungmin Kim, Lapid Maria, Joseph Gaugler, Dawne Finnie, Theresa Frangiosa

**Affiliations:** Medstar Health, Minneapolis, Minnesota, United States; Mayo Clinic, Rochester, Minnesota, United States; Seoul National University, Seoul, Republic of Korea; Mayo Clinic, Rochester, Minnesota, United States; University of Minnesota, Minneapolis, Minnesota, United States; Mayo Clinic, Rochester, Minnesota, United States; Faerge Drinker, Washington, District of Columbia, United States

## Abstract

Lucid episodes (LE) occur in people living with dementia who have seemingly lost cognitive capacity. Understanding LEs is a relatively new area of scientific inquiry. Most LE studies have utilized different conceptual and operational definitions. Reaching a definition of LE is critical foundation to facilitate more systematic investigation and new knowledge of LEs. The goal of this study was to convene a group of 13 clinical experts to brainstorm on the key components needed to understand LE among people living with dementia (PLWD). We aimed to reach consensus from the group on (1) potential medical or clinical explanations for LEs; (2) necessary medical and clinical questions to understand LE; (3) interpretation of the presence of LE. Thirteen experts from different disciplines (neurology, psychiatry, psychology, pharmacy, palliative care, hospice, nursing, social work, primary care, geriatrics, and professional home caregivers) participated in a virtual Delphi Method study. Experts provided a range of opinions on potential medical and clinical explanations for LE, necessary questions to understand LE, and did not reach consensus on the presence of LE when presented with clinical vignettes. Panelists highlighted key medical and contextual factors as well as a need to know more information about the general demeanor and typical behavior of the PLWD to make a definitive judgement about whether LE occurred. Clinical consensus on LEs is complex. There is great variability in how behaviors and cognitive fluctuations in PLWD are appraised by clinical experts.

